# Intranasal Administration of Insulin Reduces Chronic Behavioral Abnormality and Neuronal Apoptosis Induced by General Anesthesia in Neonatal Mice

**DOI:** 10.3389/fnins.2019.00706

**Published:** 2019-07-11

**Authors:** Hengchang Li, Chun-ling Dai, Jin-Hua Gu, Shengwei Peng, Jian Li, Qian Yu, Khalid Iqbal, Fei Liu, Cheng-Xin Gong

**Affiliations:** ^1^Department of Neurochemistry, New York State Institute for Basic Research in Developmental Disabilities, Staten Island, NY, United States; ^2^Department of Anesthesiology, Guangzhou First People’s Hospital, School of Medicine, South China University of Technology, Guangzhou, China; ^3^Department of Clinical Pharmacy, Nantong Maternity and Child Health Hospital, Nantong University, Nantong, China; ^4^Department of Internal Medicine, Hubei University of Science and Technology, Xianning, China; ^5^Department of Pediatrics, The Second Xiangya Hospital, Central South University, Changsha, China; ^6^Department of Orthopedic, Shandong Qianfoshan Hospital, Shandong University, Jinan, China

**Keywords:** anesthesia, apoptosis, cognitive function, intranasal administration, insulin, neonatal mice, sevoflurane

## Abstract

Children, after multiple exposures to general anesthesia, appear to be at an increased risk of developing learning disabilities. Almost all general anesthetics—including sevoflurane, which is commonly used for children—are potentially neurotoxic to the developing brain. Anesthesia exposure during development might also be associated with behavioral deficiencies later in life. To date, there is no treatment to prevent anesthesia-induced neurotoxicity and behavioral changes. In this study, we anesthetized 7-day-old neonatal mice with sevoflurane for 3 h per day for three consecutive days and found that the anesthesia led to mild behavioral abnormalities later in life that were detectable by using the novel object recognition test, Morris water maze, and fear conditioning test. Biochemical and immunohistochemical studies indicate that anesthesia induced a decrease in brain levels of postsynaptic density 95 (PSD95), a postsynaptic marker, and marked activation of neuronal apoptosis in neonatal mice. Importantly, insulin administered through intranasal delivery prior to anesthesia was found to prevent the anesthesia-induced long-term behavioral abnormalities, reduction of PSD95, and activation of neuronal apoptosis. These findings suggest that intranasal insulin administration could be an effective approach to prevent the increased risk of neurotoxicity and chronic damage caused by anesthesia in the developing brain.

## Introduction

The developing brain, especially during the period of its growth spurt from the third trimester of gestation to the age of 2–3 years in humans, is highly vulnerable to insults. Both human and animal studies have demonstrated the neurotoxicity and the risk for permanent damage to the developing brain after general anesthesia. Unfortunately, anesthesia is essential for surgical procedures. Some children even undergo several surgeries, for example, for correcting some congenital abnormalities. Children who have multiple exposures to anesthetics and surgery appear to be at an increased risk of developing learning disabilities (Wilder et al., 2009; Flick et al., 2011). Almost all general anesthetics—including sevoflurane, which is commonly used for children—are potentially neurotoxic to the developing brain (Vlisides and Xie, 2012).

Animal studies have shown that anesthesia of neonatal rodents causes disturbed neurogenesis (Stratmann et al., 2009; Zhu et al., 2010), neuroapoptosis (Yonamine et al., 2013; Pellegrini et al., 2014), neuroinflammation (Shen et al., 2013a), oxidative stress, mitochondrial dysfunction (Sanchez et al., 2011; Boscolo et al., 2012), disturbed neurotransmission (Sanchez et al., 2011; Zhang X. et al., 2016), and disturbed synaptic ultrastructure and plasticity (Xiao et al., 2016). Neonatal rodents are much more vulnerable than mature animals to brain damage induced by anesthesia (Stratmann et al., 2009). Anesthesia-induced neurotoxicity in the developing brain may also be associated with behavioral deficiencies later in life (Shen et al., 2013a,b; Yonamine et al., 2013; Lu et al., 2016; Xiao et al., 2016; Zhang X. et al., 2016), but these animal studies showed inconsistent results. To date, there is no effective treatment that can prevent anesthesia-induced neurotoxicity and behavioral deficiencies later in life.

We recently discovered that intranasal administration of insulin, which can bypass the blood-brain barrier and deliver insulin directly to the brain (Hanson and Frey, 2008), can prevent anesthesia-induced brain changes and cognitive impairment in adult and aged mice (Chen et al., 2014b, 2017; Zhang Y. et al., 2016). Several human clinical trials have shown that intranasal insulin administration is safe and improves cognitive function in healthy adults and in cognitively impaired individuals (Benedict et al., 2008; Reger et al., 2008; Krug et al., 2010; Claxton et al., 2015). The present study aimed to investigate the long-term impact of sevoflurane anesthesia in neonatal mice and to study whether intranasal insulin can prevent anesthesia-induced brain changes and behavioral deficits later in life.

In the present study, we found that intranasal administration of insulin prevented anesthesia-induced long-term behavioral abnormalities in neonatal mice. We further demonstrated that the insulin treatment prevented anesthesia-induced reduction of PSD95 and activation of apoptosis in the neonatal mouse brain.

## Materials and Methods

### Materials and Reagents

Sevoflurane was purchased from Henry Schein, Inc. (Melville, NY, United States). Insulin (Humulin R U-100) was from Eli Lily (Indianapolis, IN, United States). Primary antibodies used in this study are listed in Table 1. Peroxidase-conjugated anti-mouse and anti-rabbit IgG were obtained from Jackson ImmunoResearch Laboratories (West Grove, PA, United States). The enhanced chemiluminescence (ECL) kit was from Pierce (Rockford, IL, United States). Other chemicals were from Sigma-Aldrich (St. Louis, MO, United States) unless otherwise stated.

**TABLE 1:** Primary antibodies used in this study.

**Antibody**	**Type**	**Source (Catalog #)**
Anti-Synapsin	Polyclonal	Enzo Life Sciences, Inc. (ADI-VAP-SV060)
Anti-Synaptophysin	Monoclonal	Millipore (MAB5258)
Anti-PSD95	Monoclonal	Cell Signaling Technology (3450S)
Anti-Iba1	Polyclonal	Abcam (ab5076)
Anti-GFAP	Monoclonal (rabbit)	Steinberger (SM122)
Anti-Cleaved caspase-3	Monoclonal (rabbit)	Cell Signaling Technology (#9664)
Anti-GAPDH	Polyclonal	Sigma-Aldrich (G9545)

### Animals and Animal Treatment

The breeding pairs of C57BL/6 mice were initially obtained from Jackson Laboratory (New Harbor, ME, United States). The mice were bred in our air-conditioned animal facility and housed with a 12/12 h light/dark cycle and with *ad libitum* access to food and water. The housing, breeding, and animal experiments were approved by the Institutional Animal Care and Use Committee of New York State Institute for Basic Research in Developmental Disabilities and were in accordance with the PHS Policy on Human Care and Use of Laboratory animals (revised March 15, 2010).

Induction of anesthesia was carried out by placing neonatal mice at the age of postnatal (P) day 7 in an anesthesia chamber (25 cm × 15 cm × 13 cm) filled with 5% sevoflurane in a mixture of O_2_ and N_2_ (50/50%). The sevoflurane concentration was reduced to 3% after an induction period of ∼3 min and maintained for 3 h. The air flow rate was 0.9–1.0 L/min during anesthesia. A small Petri dish of water was placed into the anesthesia chamber for maintaining moisture. At the end of anesthesia, the sevoflurane was turned off, and the mouse pups were kept in the same chamber with O_2_ and N_2_ for 1 h to allow their recovery from anesthesia. A warm pad was placed in the anesthesia chamber to maintain the body temperature of the neonatal mice to 35–36°C during the procedure. After waking from anesthesia, the mouse pups were returned to their parents’ cages. Neonatal mice used as controls were removed from the parents’ cages and left in the chamber filled with a mixture of O_2_ and N_2_ (50/50%) but with no sevoflurane for the same periods of time as the anesthetized group. This procedure of anesthesia was repeated for two more consecutive days.

The neonatal mice received a total of 7.0 μl insulin (0.14 U/mouse) or saline treatment through intranasal delivery 30 min before the beginning of each anesthesia. The manual intranasal administration method was modified from that for adult mice reported previously (Marks et al., 2009). Briefly, the P7-9 mouse pups were held in a supine position in hand, and 1.0 μl insulin or saline was delivered into the left side nare using a 2.5 μl Eppendorf pipette. The pups were given 15–20 s to allow the fluid to be taken into the nose before repeating the administration six times.

**FIGURE 1:**
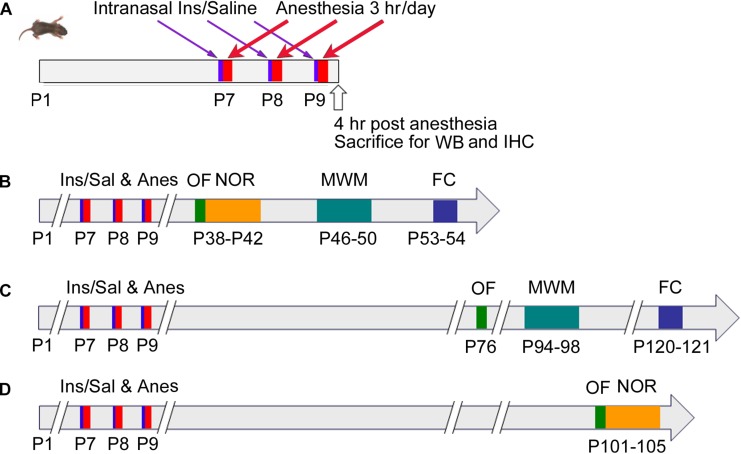
Schematic of animal studies. P7 neonatal mice were randomly assigned to cohorts **(A–D)**. In cohort **(A)**, Mice at P7 (day of birth was designed as P1) received intranasal administration of insulin, followed by 3-h anesthesia with sevoflurane starting 30 min after insulin administration. The mice were then sacrificed 4 h after the last of the 3-day insulin-anesthesia treatments for Western blots (WB) and immunohistochemical (IHC) studies. For cohorts **(B–D)**, the neonatal mice were treated as in cohort **(A)**, but they were not sacrificed and instead were tested behaviorally by using open field (OF), novel object recognition (NOR) test, Morris water maze (MWM), and fear conditioning (FC) at various postnatal ages indicated in the figure.

Neonatal mice (P7, both male and female) from various litters were randomly assigned into four groups: (1) control (Con) group that received intranasal administration of saline instead of insulin and were not anesthetized; (2) anesthesia (Anes) group that received intranasal saline followed by anesthesia with sevoflurane; (3) anesthesia plus insulin (Anes+Ins) group that received both; and (4) control insulin (Ins) group that received insulin but not sevoflurane. Mouse pups at the age of P7 with body weight less than 2.2 g were excluded from the study. At least 30 neonatal mice per group were employed so that at least 12 mice/group of each sex were available for behavioral tests. None of the behavioral tests were repeated in the same mice twice to avoid potential interference by the previous testing, so that different cohorts of mice were used when the same tests were performed at different time points post-anesthesia (Figure 1). To eliminate any potential bias caused by litter variations, similar numbers of mouse pups from each litter were assigned to each group, and each group included pups from multiple litters.

### Behavioral Tests

Behavioral tests were performed at the ages indicated in Figure 1. These tests include open field (OF) test for assessing general spontaneous activity and anxiety, novel object recognition (NOR) test for assessing memory, Morris water maze (MWM) test for assessing the hippocampus-dependent spatial learning and memory, and contextual and cued fear conditioning (FC) test for assessing learning and memory that involve the amygdala, hippocampus, frontal cortex and cingulate cortex. When several tests were performed in the same mice, the less stressful test (i.e., OF) preceded the more stressful tests (Figure 1).

The OF test was carried out by allowing mice to freely explore in an OF arena for 15 min. The testing apparatus was a classic OF (i.e., a polyvinyl chloride square arena, 50 cm × 50 cm, with walls 40 cm high), surmounted by a video camera connected to a computer. Each mouse was placed individually in the arena, and the performance was monitored and the time spent in the center and peripheral area and the distance traveled in the arena were automatically recorded by a video tracking system (ANY-Maze version 4.5 software, Stoelting Co., Wood Dale, IL, United States).

The one-trial NOR test was based on the innate tendency of rodents to differentially explore novel objects over familiar ones in an OF arena. The procedure consisted of three different phases: a habituation phase, a sample phase, and a test phase. Following initial exposure, four additional 10-min daily habituation sessions were introduced to mice for becoming familiar with the apparatus and the surrounding environment. On the fifth day, each mouse was first submitted to the sample phase, of which two identical objects were placed in a symmetric position from the center of the arena, and was allowed to freely explore the objects for 5 min. After a 15-min delay during which the mouse was returned to its home cage, the animal was reintroduced into the arena to perform the test phase. The mouse was then exposed to two objects for another 5 min: a familiar object (previously presented during the sample phase) and a novel object, placed at the same location as during the sample phase. Data collection was performed using a video tracking system (ANY-Maze version 4.5 software, Stoelting Co.). Object discrimination was evaluated by discrimination index: (time spent exploring the new object/time spent exploring both old and new objects) during the test phase.

The MWM test was performed in a white pool of 180 cm in diameter filled with water tinted with non-toxic white paint and maintained at room temperature (21 ± 2°C). During training, a platform (14 cm in diameter) was submerged 1 cm below water surface. All mice received four trials per day for four consecutive days. The starting position was randomized among four quadrants of the pool. For each trial, a mouse was allowed 90 s to locate the hidden platform. If a mouse failed to find the platform within 90 s, it was gently guided to it. At the end of each trial, the mouse was left on the platform for 20 s, then dried and returned to its home cage until the next trial. The probe trial was carried out 24 h after the last day of training. During the probe trial, mice were allowed to swim in the pool without the escape platform for 60 s. The latency to reach the platform site (seconds), number of platform location crossings, time in the target quadrant (seconds), distance covered in the target quadrant (centimeters), and swim speed (centimeters/second) were recorded using an automated tracking system (smart video tracking system, Panlab, Harvard Apparatus). The reversal water maze task was performed the next day following the standard water maze. This involved moving the location of the escape platform diagonally, followed by a 3-day acquisition phase, with a probe trial on day 4. The same parameters as for the standard MWM were recorded using the video tracking system.

For the contextual and cued FC test, mice were habituated in the testing room for one day prior to experiment. In a fear-conditioning training session, the mice were habituated to the context for 120 s, and then four tone-shock pairs consisting of a 30-s tone (2000 Hz, 75 dB) co-terminating with a 2-s foot shock at 0.6 mA delivered with a 120-s interval. Afterward, mice remained in the context for 120 s before being returned to their home cage. In the context session on day 2, mice were placed into the same testing chamber without tone or electric foot shock for 5 min to measure freezing response to the context. For the cued tone test, the mice were introduced to the same chamber with altered context. After a 180-s baseline habitation, a 180-s tone was delivered without the shock pairing, and then the mice remained in the chamber for 90 s before being returned to their home cage. All the data were collected and analyzed with the Freeze Frame and Freeze View system (Coulbourn Instruments, Whitehall, PA, United States).

### Western Blot Analysis

The mouse pups were sacrificed by decapitation, and the forebrains were removed and homogenized in pre-chilled buffer containing 50 mM Tris–HCl (pH 7.4), 50 mM GlcNAc, 20 μM UDP, 2.0 mM EGTA, 2.0 mM Na_3_VO_4_, 50 mM NaF, 20 mM Glycero-phosphate, 0.5 mM AEBSF, 10 μg/ml aprotinin, 10 μg/ml leupeptin, and 4 μg/ml pepstatin A. Protein concentrations of the homogenates were determined by using the Pierce 660-nm Protein Assay (Rockford, IL, United States). The samples were resolved by 10% SDS–PAGE and electro-transferred onto Immobilon-P membrane (Millipore, Bedford, MA, United States). The blots were then probed with primary antibodies and developed with the corresponding horseradish peroxidase-conjugated secondary antibodies and ECL kit.

### Immunohistochemistry/ Immunofluorescence

Some mouse brains were immersion-fixed in 4% paraformaldehyde at 4°C for 24 h, followed by dehydration in 30% sucrose at 4°C for 48 h. Coronal brain sections (40-μm thick) were cut by using a freezing sliding microtome. The sections were stored in antifreeze solution, consisting of glycerol, ethylene glycol and PBS at a ratio of 3:3:4 and at −20°C until immunofluorescence staining at a later time.

Coronal mouse brain sections at the same level, as evidenced by the identical hippocampal size and structure in the sections, were chosen for immunofluorescence studies. The brain sections were first washed with phosphate-buffered saline (PBS) three times, 15 min each, followed by incubation in 0.5% Triton X-100 in PBS for 20 min. The sections were then washed with PBS for another 10 min and blocked in PBS containing 5% normal goat serum and 0.1% Triton X-100 for 30 min, followed by incubation with antibody against cleaved caspase-3 at 4°C overnight. After washing with PBS again, the sections were incubated with Alexa 488-conjugated goat anti-mouse IgG (1:1000) at room temperature for 2 h. The sections were finally washed, mounted, and cover slipped using Prolong^®^ gold anti-fade mountant (Invitrogen, Carlsbad, CA, United States). The immunostaining was analyzed by using a laser scanning confocal microscope (PCM 200, Nikon). The immuno-positive cells were counted manually from three sections per mouse brain and six brains per group were calculated.

### Statistical Analysis

The quantitative data were analyzed using appropriate statistical tests, including one-way ANOVA for OF data, NOR data, Western blot data and immunofluorescence data, and two-way repeated measures ANOVA plus *post hoc* test for MWM data and FC data by using Graphpad. All data are presented as means ± SEM, and *p* < 0.05 was considered statistically significant.

## Results

### General Anesthesia With Sevoflurane in Neonatal Mice Induces Mild Long-Term Behavioral Abnormalities

Previous studies reported inconsistent results on the impact of a single anesthetic treatment of neonatal mice on behavior assessed later in life (Shen et al., 2013a,b; Yonamine et al., 2013; Lu et al., 2016; Xiao et al., 2016; Zhang X. et al., 2016). Repeated exposure to anesthesia appears to yield more consistent behavioral deficits that are detectable months later. Thus, we anesthetized P7 neonatal mice with sevoflurane for 3 h per day for 3 consecutive days and then studied the behavioral changes at various time points from 38 to 121 days after anesthesia exposure (Figure 1). The mouse behavior was evaluated by the OF test for assessing general spontaneous activity and anxiety, NOR test for assessing memory, MWM test for assessing the hippocampus-dependent spatial learning and memory, and contextual and cued FC test for assessing the learning and memory that involve amygdala, hippocampus, frontal cortex and cingulate cortex. The OF test was carried out at three time points (at the ages of 38, 76, and 101 days, which represent 28, 66, and 91 days post-anesthesia, respectively) and did not reveal any significant difference between mice with and mice without prior exposure to anesthesia (Supplementary Figure 1), suggesting that exposure of neonatal mice to anesthesia with sevoflurane does not have a chronic effect on spontaneous activity and anxiety level.

**FIGURE 2:**
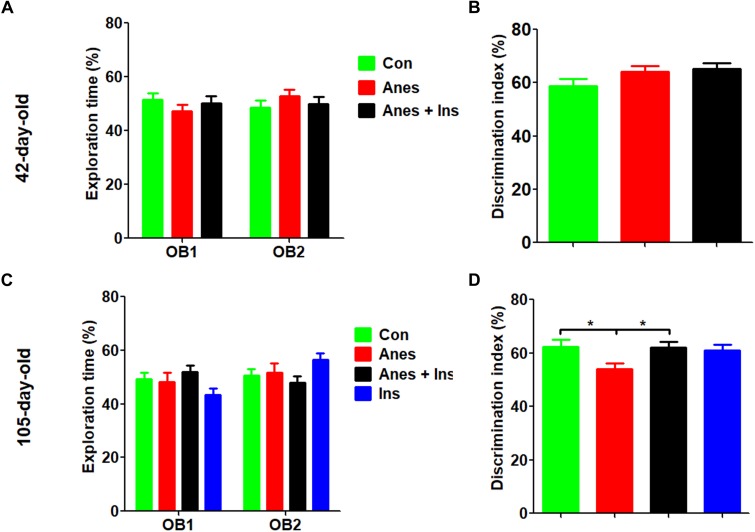
Novel object recognition test after intranasal administration and anesthesia in mice. P7 neonatal mice were anesthetized with 3% sevoflurane for 3 h/day for three consecutive days. Insulin or, as a control, saline was administered via the nasal cavity 30 min before every anesthesia. The mice were then tested in an open field for novel object recognition at the age of 42 days **(A,B)** or 105 days (female only, **C,D**). Panels **(A,C)** show the exploration times of mice during the sample phase. Panels **(B,D)** show the discrimination index during the test phase. *N* = 10–13 mice per group, ^*^p < 0.05.

The NOR test was done at 1 and 3 months after the mice were exposed to anesthesia using separate cohorts of mice (See Figure 1). We found that, as expected, mice at both time points showed similar exploration times to each object during the sample phase (Figures 2A,C). The test phase showed a mild but significant decrease in the discrimination index in female mice that were exposed to anesthesia at the age of P7-9 as compared to those not exposed to anesthesia, when they were tested at the age of 105 days (Figure 2D) but not at 42 days (Figure 2B). These results suggest that anesthesia of neonatal mice leads to a mild memory impairment that is detectable 3 months after exposure to anesthesia.

**FIGURE 3:**
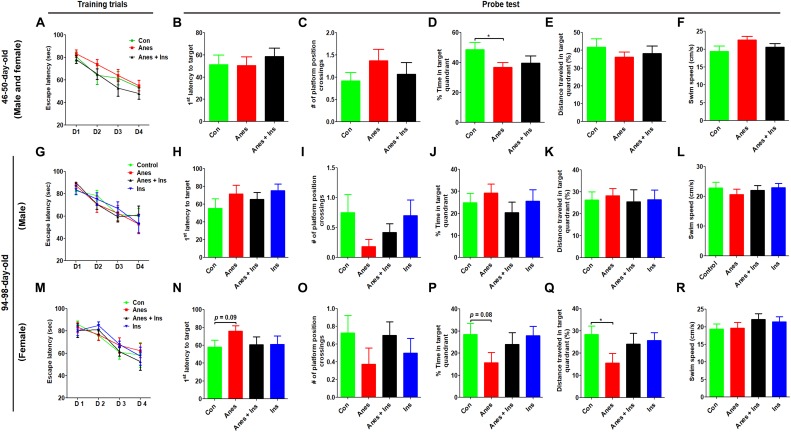
Morris water maze test after intranasal administration and anesthesia in mice. P7 neonatal mice were anesthetized with 3% sevoflurane for 3 h/day for three consecutive days. Insulin or, as a control, saline was administered via the nasal cavity 30 min before every anesthesia treatment. The mice were then tested in a Morris water maze for spatial learning and memory at the age of 40–45 days **(A–F)** or 94–98 days **(G–R)**. *N* = 10–13 mice per group, ^*^p < 0.05.

Morris water maze was used for testing spatial learning and memory at the ages of 46–50 days and 94–98 days in two cohorts of mice (see Figure 1). We did not find any significant change in the spatial learning in mice that were exposed to anesthesia during P7-9 at either age (Figures 3A,G,M). The probe test indicated that the mice previously exposed to anesthesia had significant reduction of the time spent in the target quadrant at the age of 50 days (Figure 3D) and the distance traveled in the target quadrant at the age of 98 days (Figure 3Q). The trend of increased latency to reach the target (Figures 3H,N), decreased platform position crossings (Figures 3I,Q), and decreased time spent in the target quadrant (Figure 3P) were obvious in mice previously exposed to anesthesia, though these changes did not reach statistical significance. The swim speed of these two groups of mice were not different (Figures 3F,L,R), which otherwise could contribute to the above observed changes. We also analyzed the data of male and female mice separately and found no difference between male and female mice when tested at the age of 46–50 days. Thus, we combined the data of two genders (Figures 3A–F). Interestingly, the anesthesia-induced spatial memory impairment is more apparent in female than male mice when tested at the age of 94–98 days (Figures 3G–R). Taken together, these results suggest that exposure to anesthesia with sevoflurane leads to mild spatial memory impairments that are detectable 1–3 months later and that the chronic changes might be more obvious in female than in male mice.

**FIGURE 4:**
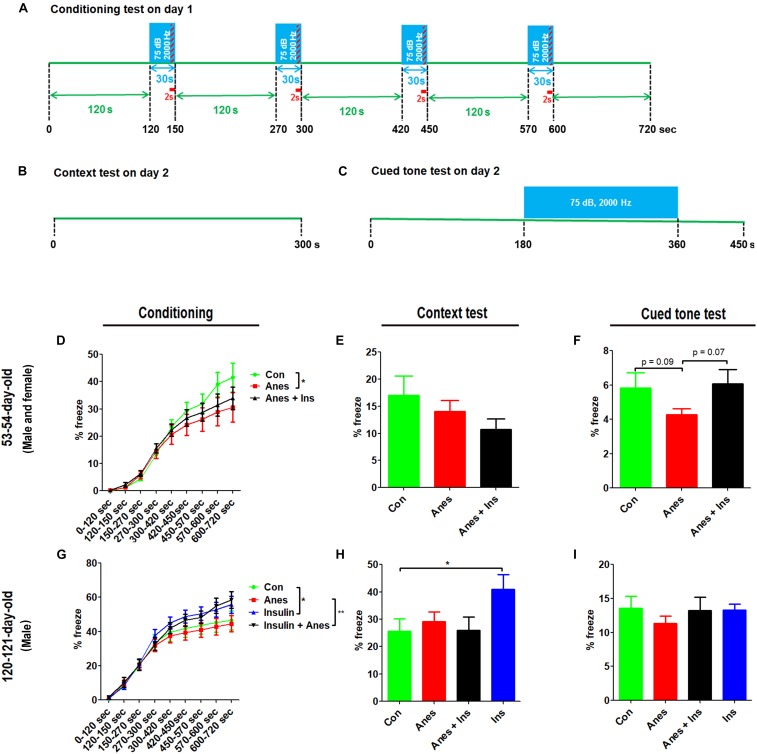
Fear conditioning test after intranasal administration and anesthesia in mice. Neonatal mice (P7 to P9) were anesthetized with 3% sevoflurane for 3 h/day for three consecutive days. Insulin or, as a control, saline was administered via the nasal cavity 30 min before every anesthesia treatment. The mice were then subjected to fear conditioning test at adult age. **(A–C)** Fear conditioning test schedule, which was carried out over two consecutive days. **(D,G)** Percentage of time the mice froze during the conditioning phase on day 1 of the FC test. **(E,H)** Percentage of time the mice froze during the first 6 min (data of 0.25 s bout) in the context test. **(F,I)** Percentage of time the mice froze (data of 0.25 s bout) during the first 7.5 min in the cued tone test results. Data are presented as mean ± SEM (*n* = 10–13 mice per group), ^*^p < 0.05.

The contextual and cued FC test was performed at the ages of 53–54 days (see Figure 1, cohort B) and 120–121 days (cohort C). The test consisted of a conditioning phase on day 1 and a testing phase on day 2 (Figures 4A–C). During the conditioning phase, mice with prior exposure to anesthesia showed significant decrease in freezing time as compared to the control mice at both ages (Figures 4D,G). No significant differences in the freezing time were seen during the context test between mice with and mice without prior exposure to anesthesia (Figures 4E,H). However, clear reductions in the freezing time were observed in the mice with prior exposure to anesthesia in the cued tone test (Figures 4F,I), though these reductions did not reach statistical significance. Taken together, these results suggest that anesthesia of neonatal mice at P7-9 with sevoflurane causes mild impairment in learning and memory associated with the amygdala, hippocampus, frontal cortex and cingulate cortex later in life.

### Intranasal Administration of Insulin Prevents Anesthesia-Induced Long-Term Cognitive Impairment in Mice

To investigate whether insulin can prevent anesthesia-induced cognitive impairment observed later in life, we included a group of mice that received intranasal administration of insulin prior to anesthesia at the age of P7-9 and another control group that received insulin but were not exposed to anesthesia. We found that like the exposure to anesthesia with sevoflurane, insulin administration did not have any significant effect on spontaneous activity and anxiety, as determined by the OF test (Supplementary Figure 1). However, the prior treatment of neonatal mice with intranasal insulin effectively prevented the anesthesia-induced reduction of the discrimination index determined by the NOR test carried out at the age of 105 days (Figure 2D). Insulin itself had no significant effect on the mice without prior exposure to anesthesia in this test. Furthermore, intranasal insulin treatment was found to prevent almost all the abnormal trends induced by prior exposure to anesthesia in the Morris water maze test, including the impaired learning curve (Figures 3A,M) and the probe test (Figures 3N–Q). Similarly, a clear protective effect of intranasal insulin was also observed by using the contextual and cued FC tests (Figures 4D,F,G). It is interesting to note that intranasal insulin administration at the age of P7-9 was found to improve the learning and memory of the control mice tested 3 months later in the FC test, as these mice showed a significantly better learning curve (Figure 4G) and longer freezing than the untreated control mice in the context test (Figure 4H). Taken together, these results suggest that the chronic mild behavioral impairments, which were detectable at adult age but caused by anesthesia with sevoflurane during the neonatal age, can be prevented by intranasal administration of insulin prior to exposure to anesthesia.

### General Anesthesia With Sevoflurane Promotes Synaptic Changes and Neuronal Apoptosis in the Brains of Neonatal Mice

**FIGURE 5:**
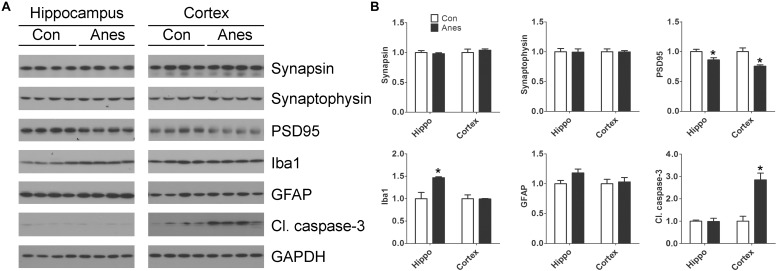
Effects of repeated anesthesia with sevoflurane on synaptic proteins, glial markers, and apoptosis marker in neonatal mouse brains. P7 mice were anesthetized with 3% sevoflurane for 3 h/day for three consecutive days and sacrificed 4 h after the last anesthesia. The hippocampi and the cerebral cortices of the mouse brains were homogenized and analyzed by Western blots developed with antibodies indicated at the right side of the blots **(A)**. Quantification of the blots are shown in panel **(B)**. ^*^p < 0.05 vs. control (*n* = 8 mice per group).

Previous studies suggest that anesthesia of neonatal mice may induce changes of synaptic proteins, neuroinflammation, and neuronal apoptosis in the brain, depending on the anesthetics and regimens (Shen et al., 2013a; Yonamine et al., 2013; Pellegrini et al., 2014; Xiao et al., 2016). To study whether the repeated anesthesia with sevoflurane to neonatal mice for three consecutive days could result in these brain changes, we determined two presynaptic proteins (synapsin and synaptophysin), one post-synaptic protein (PSD95), two markers of neuroinflammation (GFAP and Iba1), and a marker of apoptosis (cleaved/activated caspase-3) in mouse cerebral cortex and hippocampus by Western blots. We found significant reduction of PSD95, but not of synapsin or synaptophysin, both in the cerebral cortex and the hippocampus after exposure to sevoflurane (Figure 5). A marked elevation of Iba1 was observed with sevoflurane exposure in the hippocampus, but not in the cerebral cortex. A trend whereby astrocyte marker GFAP was increased was also observed in the hippocampus but not in the cortex of the mouse brains post-exposure to sevoflurane. In contrast to the microglial marker Iba1, a remarkable increase in the level of cleaved caspase-3 was seen in the cortex but not in the hippocampus of the sevoflurane-exposed mice. These results indicate brain region-specific changes of synaptic proteins, neuroinflammation markers and apoptosis in neonatal mice after exposure to anesthesia with sevoflurane.

### Intranasal Administration of Insulin Prevents Anesthesia-Induced Reduction of PSD95 and Activation of Apoptosis in the Cerebral Cortex in Neonatal Mice

**FIGURE 6:**
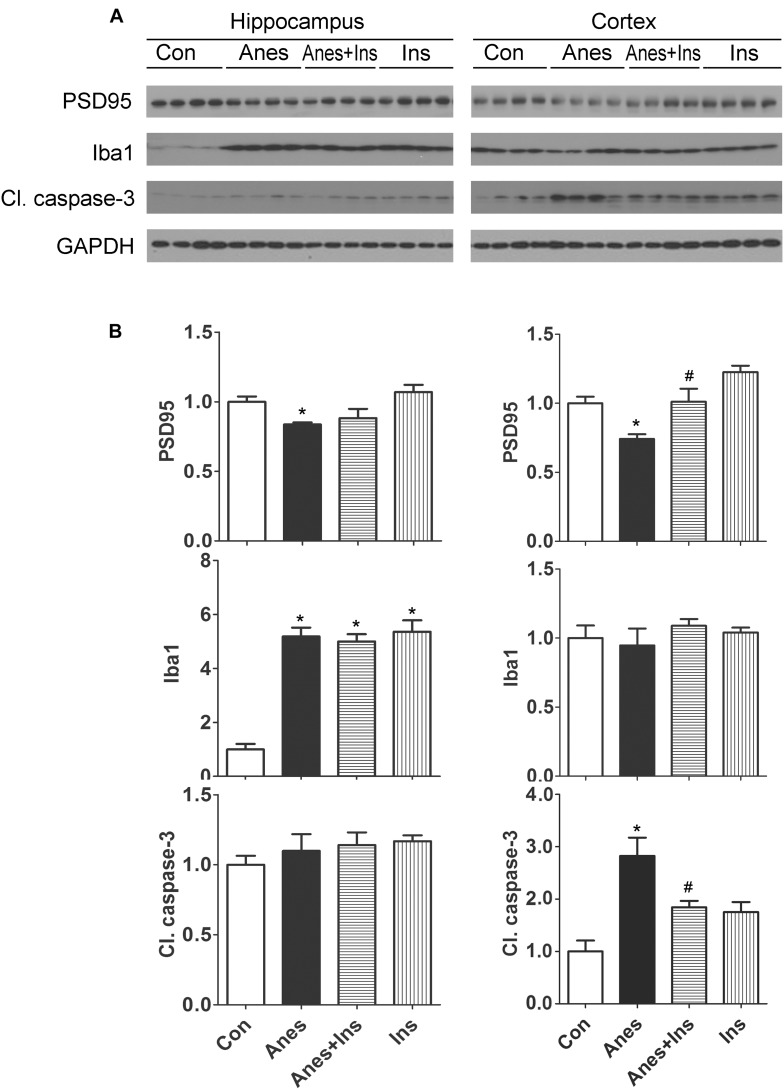
Effect of intranasal insulin on PSD95, Iba1, and apoptosis in neonatal mouse brains after repeated anesthesia with sevoflurane. P7 neonatal mice received intranasal administration of insulin (0.14 U/mouse) or, as a control, saline, followed by inhalational anesthesia with 3% sevoflurane for 3 h beginning 30 min after intranasal administration. The same treatments were repeated for two more consecutive days, followed by sacrifice at 4 h after the last anesthesia. The hippocampi and the cerebral cortices of the mouse brains were then homogenized and analyzed by Western blots **(A)**. Quantification of the blots are shown in panel **(B)**. ^*^p < 0.05 vs. control; #*p* < 0.05 vs. Anes group. *N* = 8 mice per group.

We investigated if the anesthesia-induced changes in brain PSD95, Iba1 and apoptosis can be prevented by prior intranasal administration of insulin, so we repeated the above experiments and also included two more groups (mice pretreated with insulin before anesthesia and mice treated with insulin without anesthesia) and determined the levels of these proteins in the brains. We found that prior treatment with intranasal insulin prevented the sevoflurane-induced reduction of PSD95 and increase cleaved caspase-3 levels, but it had no effect on sevoflurane-induced elevation of Iba1 level (Figure 6). Intranasal insulin alone did not induce any significant changes in the brain levels of PSD95, Iba1, or cleaved caspase-3. These results suggest that pretreatment of neonatal mice with intranasal insulin before anesthesia with sevoflurane can prevent the anesthesia-induced alterations of synaptic protein PSD95 and apoptosis.

**FIGURE 7:**
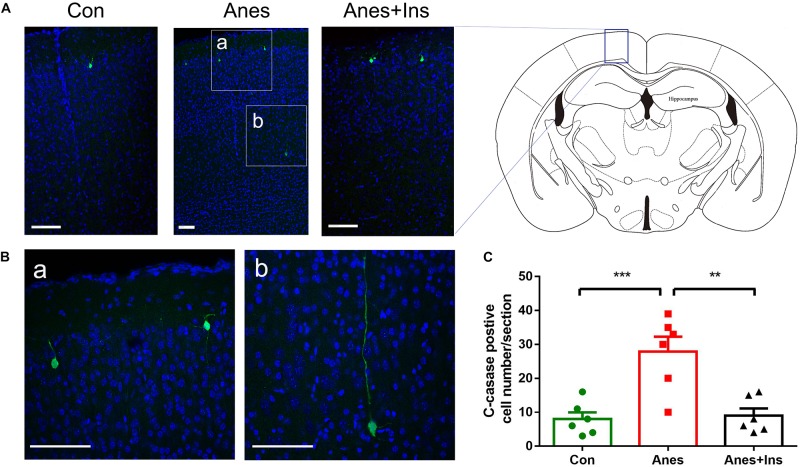
Immunohistochemical staining of brains for apoptosis after repeated anesthesia and intranasal insulin treatment in neonatal mice. **(A)** P7 neonatal mice received intranasal administration of insulin (0.14 U/mouse) or, as a control, saline, followed by inhalational anesthesia with 3% sevoflurane for 3 h beginning 30 min after intranasal administration. The same treatments were repeated for two more consecutive days, followed by sacrifice at 4 h after the last anesthesia. The mouse brain sections were immuno-stained with antibody against cleaved caspase-3 (green) and counter-stained with nuclear marker TO-PRO (blue). **(B)** The enlarged micrographs from areas marked with a and b in panel **(A)** are shown. **(C)** Neurons positive for cleaved caspase-3 in brain sections were calculated and are shown as mean ± SEM (*n* = 6 mice per group). ^∗∗^*p* < 0.01 and ^∗∗∗^*p* < 0.001 by *t*-test. Scale bars, 100 μm.

Immunofluorescence staining of the frozen coronal brain sections with antibody against cleaved caspase-3 was used to detect the sevoflurane-induced activation of apoptosis and its topographical distribution as well as the preventive effect of intranasal insulin. In the control brains, we observed only a few cleaved caspase-3-positive cells that were mainly distributed in the cerebral cortex (Figure 7A). We found approximately four-fold more cleaved caspase-3-positive cells in the brains of mice after sevoflurane exposure, and these cells were mainly distributed in the cerebral cortex (Figures 7A,C). A close examination of these cells under high magnification suggested that most, if not all, of these cleaved caspase-3-positive cells were neurons on the basis of their size and morphology (Figure 7B). Significantly fewer cleaved caspase-3-positive cells were observed in sections of mice that received pre-treatment with intranasal insulin than those without insulin treatment (Figure 7). The number of the cleaved caspase-3-positive cells in mice with insulin treatment was almost the same as those of the control brains. These results revealed that repeated anesthesia of P7 neonatal mice with sevoflurane induced neuronal apoptosis, especially in the cerebral cortex, and that intranasal insulin administered prior to anesthesia prevented the activation of apoptosis.

## Discussion

General anesthesia is essential for performing surgeries, including pediatric surgeries, in modern medical practice. Repeated anesthesia/surgeries are sometimes needed for a child, such as for correcting some congenital heart defects. The potential adverse impact of general anesthesia on children, especially infants, has been of concern recently (Servick, 2014; Andropoulos, 2018). Long-term effect of general anesthesia exposure during the early development is also suspected, but this aspect is currently at the early stage of investigation (Shen et al., 2013a,b; Yonamine et al., 2013; Lu et al., 2016; Xiao et al., 2016; Zhang X. et al., 2016). In the present study—by using the NOR test, Morris water maze and FC test—we found that anesthesia of 7- to 9-day-old mice with sevoflurane for 3 h per day for three consecutive days led to mild behavioral abnormalities that were detectable when the animals grew to adult age. Our findings of the reduction of PSD95 and over-activation of neuronal apoptosis in the mouse brain after repeated anesthesia suggest that general anesthesia might cause the behavioral changes through interruption of synaptic plasticity and neuronal apoptosis during brain development. Importantly, we found that insulin administered through intranasal delivery prior to anesthesia can prevent anesthesia-induced long-term behavioral abnormalities, reduction of PSD95, and over-activation of neuronal apoptosis.

Individuals of different ages may be vulnerable differently to general anesthesia, and children are believed to be more vulnerable than adults (Bang, 2015). Animal studies have demonstrated acute neurotoxicity in neonatal mice after exposure to anesthesia, such as disturbed neurogenesis (Stratmann et al., 2009; Zhu et al., 2010), neuroapoptosis (Yonamine et al., 2013; Pellegrini et al., 2014), neuroinflammation (Shen et al., 2013a), oxidative stress and mitochondrial dysfunction (Sanchez et al., 2011; Boscolo et al., 2012), disturbed neurotransmission (Sanchez et al., 2011; Zhang X. et al., 2016), and disturbed synaptic ultrastructure and plasticity (Xiao et al., 2016). Most of the animal studies for developing brains used P7-8 mice because this age of mice represents the period of the brain growth spurt, a critical stage of neural development, and is approximately equivalent to the first 2 years of life in humans (Semple et al., 2013). Brain during the growth spurt period is also more vulnerable to insults that could cause neuropsychological abnormalities later in life. Chronic behavioral impairments are also reported to be detectable in adult mice after their exposure to anesthesia during the neonatal age (Shen et al., 2013a,b; Yonamine et al., 2013; Lu et al., 2016; Xiao et al., 2016; Zhang X. et al., 2016). However, several other studies failed to replicate the chronic behavioral impairments (Feng et al., 2012; Murphy et al., 2017). The inconsistency among these studies may result from different species/strains, anesthetic agents, and/or anesthesia regimens used. By using three different cognitive tests, we detected only mild, though clear and significant, behavioral impairment in adult mice who were exposed to sevoflurane for 3 days, 3 h each day, at the age of postnatal day 7 to day 9. The behavioral impairment was detectable in mice from the age of 40 days to 4 months, which is approximately equivalent to 15–25 years old in humans (Semple et al., 2013). It is not surprising that these mild changes could be missed by some studies if the mice were exposed to different anesthetics and/or for a shorter time.

The structural basis of brain function is synaptic connection. The integrity of synaptic connection requires normal synapses and their structures. One indirect but easy way to study synapses is to determine if there are any changes of synaptic proteins. We therefore studied two presynaptic proteins (synapsin and synaptophysin) and one postsynaptic protein (PSD95) in the mouse brains and found that PSD95 was significantly decreased after mice were exposed to anesthesia. These results suggest alterations of postsynaptic structure and/or function that might underlie the behavioral impairment detected later in these mice. Our observations that the pre-treatment of neonatal mice with intranasal insulin prevented anesthesia-induced reduction of PSD95 suggest that the insulin’s protection against anesthesia-induced behavioral impairment might partially result from its protection on synaptic integrity. It has been reported previously that insulin can regulate synaptic density (Chiu et al., 2008) and increase the expression of postsynaptic receptors, such as GABA(A) receptors, on the postsynaptic and dendritic membranes (Wan et al., 1997).

Over-activation of neuronal apoptosis was one of the most remarkable changes we found in the brains of mice after exposure to anesthesia. This over-activation could alter neural development and thus also contribute to the behavioral impairment detected at later dates. It is currently unknown whether the reduction of PSD95 is associated with the over-activation of neuronal apoptosis after anesthesia exposure. A recent study also found decreased PSD95 level and activation of apoptosis in the brains of adult rats after exposure to sevoflurane (Lu et al., 2017). Reduction of PSD95 and activation of apoptosis in the brain have been reported as well in individuals with mild cognitive impairment and with Alzheimer’s disease (Sultana et al., 2010).

Different types of anesthetics might have different adverse impacts on the developing brain. We chose sevoflurane as the anesthetic agent in the present study because it is among the most commonly used pediatric anesthetics in the current medical practice, and thus our findings are of direct clinical relevance. Sevoflurane and its derivative, isoflurane, are also often used for studies to investigate neurotoxicity of inhalational anesthetics. Neurotoxicities of other types of anesthesia agents on the developing brain have also been reported (Davidson, 2016). Therefore, we speculate that intranasal insulin may also have similar preventive benefits against neurotoxicities and adverse functional changes induced by other anesthetics. This speculation will eventually need to be confirmed by future studies.

Recent studies suggest that insulin has neurotrophic and neuroprotective activities, regulates neural development and plasticity, and plays an important role in learning and memory (Ghasemi et al., 2013; Blazquez et al., 2014; Chen et al., 2014a). Insulin in the brain appears to be derived both from the periphery via receptor-mediated transport (Miller et al., 1994) and from its synthesis by some neurons in the brain (Schechter et al., 1988; Devaskar et al., 1994). However, it is challenging to administer insulin into the brain because the peripheral administration may lead not only to hypoglycemia, but also to a very limited amount entering the brain. The intranasal delivery of insulin, which was invented by W.H. Frey II of Regions Hospital in St. Paul, MN (Hanson and Frey, 2008; Jogani et al., 2008), appears to be an effective and practical method for delivering insulin directly into the brain without significant hypoglycemia. This delivery technique has been tested successfully both in animal studies by us and others (Chen et al., 2014c, 2017; Zhang Y. et al., 2016) and in human clinical trials (Benedict et al., 2008; Reger et al., 2008; Krug et al., 2010; Claxton et al., 2013). Thus, we adapted this technique for the delivery of insulin into the neonatal mouse brain. Our findings of the improved cognitive functions and the prevention of PSD95 reduction and neuronal apoptosis after intranasal insulin treatment indicate the successful delivery of insulin into the neonatal mouse brain through intranasal administration.

It remains to be studied how insulin can prevent anesthesia-induced damage in the developing brain. Insulin has multiple functions in the brain, including neurotrophic activity, and it regulates neural development and plasticity (Ghasemi et al., 2013; Blazquez et al., 2014; Chen et al., 2014a). It is possible that these functions of insulin may underlie its beneficial effect we observed in the present study because anesthesia might damage the developing brain through disruption of neurotrophics and neurodevelopment (Wu et al., 2016; Gui et al., 2017; Liu et al., 2017). We recently found that general anesthesia disturbs, and that intranasal insulin promotes, brain insulin signaling in the adult mouse brains (Chen et al., 2014c). Intranasal insulin has been shown to promote neuronal energy metabolism. A brain imaging study using ^31^P-MRI found increased brain cell ATP and phosphocreatine, as well as suppressed food intake, after intranasal insulin treatment in humans (Jauch-Chara et al., 2012). Thus, the role of intranasal insulin in enhancing brain energy metabolism during anesthesia may contribute to its neuroprotection against anesthesia-induced neurotoxicity. Anesthesia and sedation can cause neuronal stress and activate stress response. Elevation of salivary cortisol levels is observed in children after routine sedated procedures (Hsu et al., 2012). Interestingly, intranasal insulin can attenuate the hypothalamic-pituitary-adrenal axis response to stress in human (Bohringer et al., 2008). Since cortisol greatly inhibits the uptake of glucose into the hippocampus (Frey, 2013), intranasal insulin can therefore reduce anesthesia-induced increase in cortisol and, in turn, result in increased brain cell energy.

A recent study found that intranasal insulin improves memory, increases cerebral glucose uptake, and decreases neuroinflammation and hippocampal lesion volume caused by traumatic brain injury in rats (Brabazon et al., 2017). The anti-inflammation action of insulin has previously been demonstrated in many studies (Dandona et al., 2007; Sun et al., 2014). Therefore, anti-inflammation might also be involved in insulin’s neuroprotective effects and the prevention of anesthesia-induced behavioral impairment we observed, although we did not observe the corresponding changes of microglial marker Iba1 at the time point selected in the present study.

Only a few studies investigated potential strategies aimed at preventing anesthesia-induced neurotoxicity and behavioral deficits in animals. Co-administration of hydrogen gas as part of the carrier gas mixture was found to suppress neuroapoptosis and subsequent behavioral deficits caused by neonatal exposure to sevoflurane in mice (Yonamine et al., 2013). Treatment of newborn rats with EUK-134 or R(+)-pramipexole, which reduces free oxygen radicals and restores mitochondrial integrity, reduces anesthesia-induced neuronal loss (Boscolo et al., 2012). Anti-inflammatory treatment of neonatal mice may ameliorate sevoflurane-induced cognitive impairment (Shen et al., 2013a). Administration of erythropoietin, a potent neuroprotective agent, immediately after postnatal exposure to sevoflurane reduces both activation of neural apoptosis and cognitive impairment at a later age (Pellegrini et al., 2014). However, there are no follow-up studies reported after these initial investigations, and the safety concerns of these treatments for children remain elusive.

The developing brain, especially during the period of the brain’s growth spurt from the third trimester of gestation to the age of 2–3 years in humans, is highly vulnerable to insults. Both human and animal studies have suggested the neurotoxicity and risk for permanent damage to the developing brain with anesthesia. Considering that 6 million children under the age of 15 years, including 1.5 million infants under 12 months, receive anesthesia in the United States alone each year (Sun et al., 2008; Servick, 2014), developing a treatment to prevent or reduce anesthesia-induced neurotoxicity and long-term brain damage has become very urgent. Our findings of the neuroprotective effects of intranasal insulin provide initial indication for developing a simple preventive treatment against anesthesia-induced brain damage in children.

## Data Availability

The datasets generated for this study are available on request to the corresponding author.

## Ethics Statement

The housing, breeding, and animal experiments were approved by the Institutional Animal Care and Use Committee of New York State Institute for Basic Research in Developmental Disabilities and were in accordance with the PHS Policy on Humane Care and Use of Laboratory animals (revised March 15, 2010).

## Author Contributions

C-XG, FL, and KI designed the research. HL, C-lD, J-HG, SP, JL, and QY performed the experiments. C-XG, HL, C-lD, and J-HG analyzed the data. HL and C-XG wrote the manuscript. All authors contributed to the approval of the final manuscript.

## Conflict of Interest Statement

KI serves on the scientific advisory board of AXON Neuroscience, has received research grants from Ever NeuroPharma and Signum Biosciences, and holds several patents on treatment of Alzheimer disease and related conditions. C-XG serves on the scientific advisory board of Alectos Therapeutics. KI, FL, and C-XG hold a patent on intranasal insulin administration for the minimization of anesthesia-induced memory loss. The remaining authors declare that the research was conducted in the absence of any commercial or financial relationships that could be construed as a potential conflict of interest.
