# Supplementary material for: Intranasal Administration of Insulin Reduces Chronic Behavioral Abnormality and Neuronal Apoptosis Induced by General Anesthesia in Neonatal Mice
Source: Front Neurosci. 2019 Jul 11;13:706. doi: 10.3389/fnins.2019.00706 (PMC6637386; doi:10.3389/fnins.2019.00706)
Supplement: Supplementary file 1 [file Table_1.DOCX]

**Supplementary Figure 1:**

**
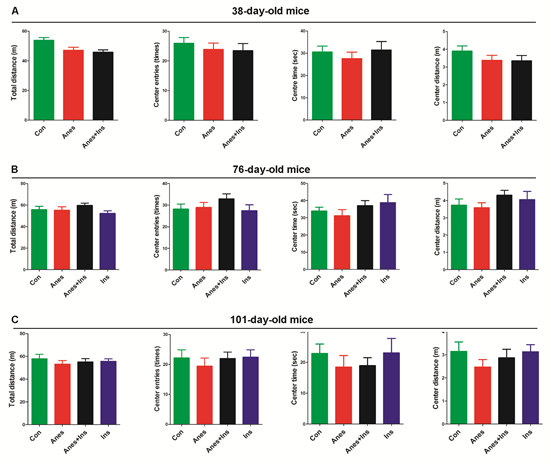
**

**Supplementary Fig. 1. Open field test after intranasal administration and anesthesia in mice.** P7 neonatal mice were anesthetized with 3% sevoflurane for 3 hrs/day for three consecutive days. Insulin or, as a control, saline was administered via nasal cavity 30 min before every anesthetic treatment. The mice were then tested in an open field at the age of 38 days (A), 76 days (B), or 101 days (C). The total distance the mice traveled, the number of center entries, the time spent and the distance covered in the center of the arena were recorded. N=10–13 mice per group.
